# Recurrent respiratory papillomatosis: the role of cidofovir

**DOI:** 10.1002/rcr2.371

**Published:** 2018-10-02

**Authors:** Mai Ngoc Tran, Lauren Galt, Farzad Bashirzadeh

**Affiliations:** ^1^ Thoracic Medicine Department The Royal Brisbane and Woman’s Hospital Brisbane Australia

**Keywords:** Cidofovir, chronic condition, human papilloma virus, recurrent respiratory papillomatosis, remission

## Abstract

Recurrent respiratory papillomatosis (RRP) is a rare condition that affects the respiratory system. It is caused by human papilloma virus (HPV) infection. Usually infection and papilloma growth is limited to 6–12 months duration; however, some patients have persistent disease, resulting in long‐term symptoms and the need for recurrent intervention. Predominant symptoms include shortness of breath, reduced exercise tolerance and voice deterioration during flares. Current gold‐standard management is through resection via microdebrider, CO_2_ laser, cryotherapy, electrocoagulation, Nd: YAG laser or pulse‐dye laser. However, despite these therapies, approximately 20% of patients require adjuvant therapy. We discuss the use of intralesional cidofovir in the management of tracheal papillomatosis. Cidofovir’s mechanism of action involves incorporating into the virus DNA chain and therefore, inhibiting the viral DNA polymerization process and hence replication.

## Introduction

Recurrent respiratory papillomatosis (RRP) is a difficult entity to treat. A robust evidence‐based efficacious treatment regime to achieve remission for this debilitating condition remains elusive. This case study describes the use of intralesional cidofovir in successfully prolonging symptom remission of RRP.

## Case Report

A 42‐year‐old male who was exposed to human papilloma virus (HPV) at birth developed chronic upper airway papillomatosis. He was exclusively managed by ear, nose, and throat surgeons (ENT) from 20 months old (first manifestation) until 42 years of age, when his papillomatosis disease extended to involve his upper trachea. At this time, in 2013, he was referred to our respiratory service for management.

Past medical history included a current 22 pack‐year smoking history, with no other comorbid medical conditions. There is no family history or personal history of immunodeficiency. He worked as a shopkeeper and had no other known exposures.

**Figure 1:**
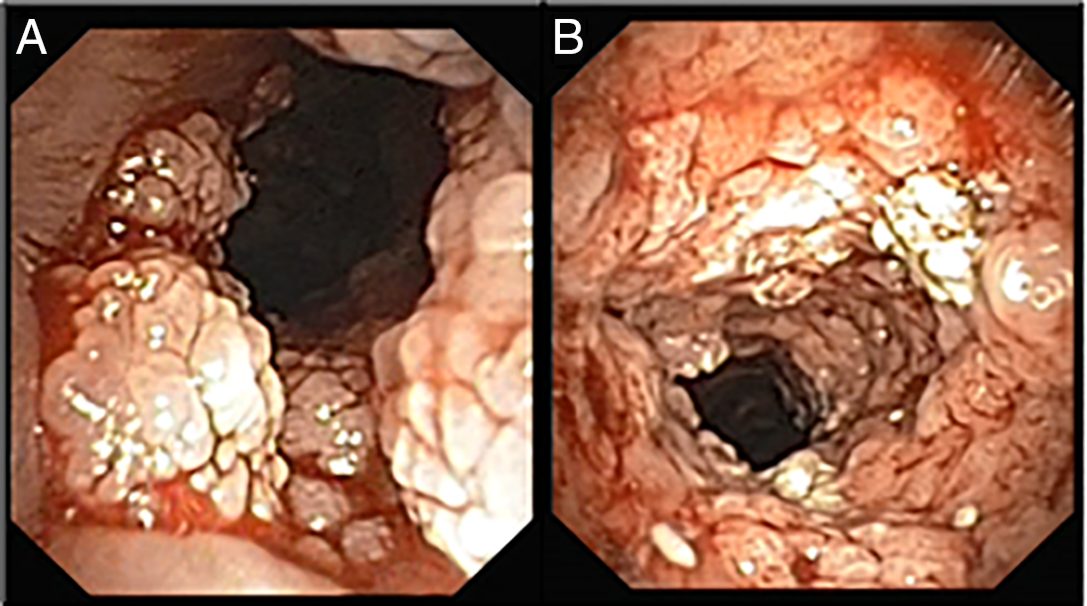
Disease burden pre‐administration of intralesional cidofovir. (A) Large papillomas immediately below the vocal cords before treatment. (B) Papillomas in the proximal trachea after removal of papillomas but before injection with cidofovir.

The patient developed marked symptoms as the papillomas grew. These included: constant shortness of breath, reduced exercise tolerance, cough, wheeze, hoarse and quiet voice. The severity of these symptoms resulted in him being unable to work due to poor voice projection. On examination, auscultation revealed bilateral polyphonic expiratory wheeze and monophonic inspiratory wheeze. Direct visualization via bronchoscopy demonstrated significant HPV polyposis of the larynx, vocal cords, supraglottis, subglottis, 40–50% of his trachea involved and significant narrowing of airways (see Fig. 1). The distal trachea was not affected.

He has had 166 exacerbations requiring intervention for regrowth of polyps. Between 2013 and 2015, he was managed with numerous multiple laser ablation procedures via mircolaryngoscopy and flexible bronchoscopy. Previously tried therapies included: argon plasma coagulation (APC), topical mitomycin, CO_2_ laser, diathermy snare, electrocautery ablation, dietary changes and natural remedies. The histopathology of the resected respiratory papillomas demonstrated squamous papillomas with focal koilocytic change. There was no evidence of high‐grade dysplasia or malignancy in resected specimens.

**Figure 2:**
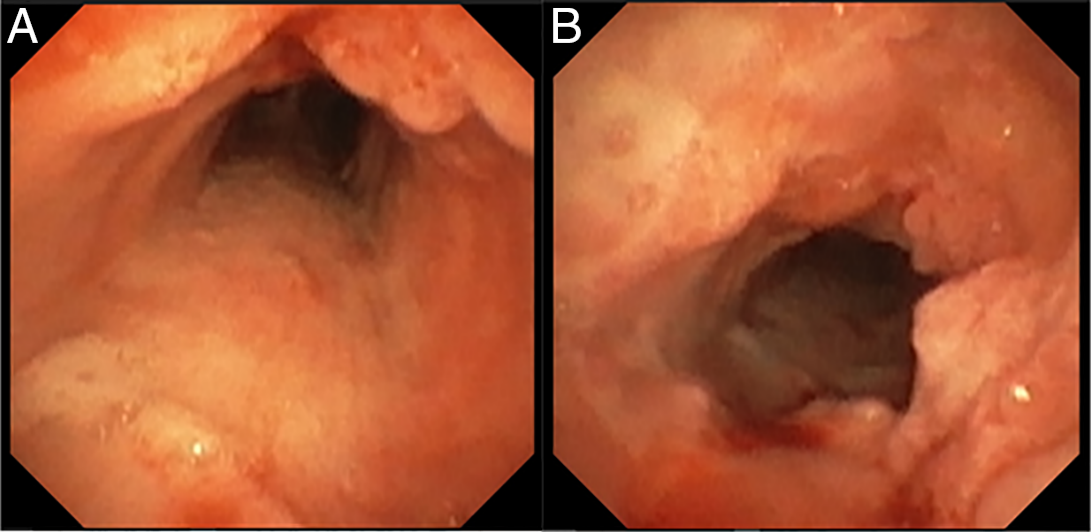
Disease burden on final follow up after two sets of intralesional cidofovir injections. (A) Disease burden below cords to mid trachea at 6 month follow up after the second set of cidofovir injections. (B) Disease burden in the proximal trachea after second treatment round of intralesional cidofovir.

In 2016, a decision was made to use intralesional cidofovir, with the aim of achieving better disease control and reducing the frequency and severity of exacerbations. Fibre‐optic bronchoscopy facilitated the removal of the papillomas via pulsed APC, a circumferential catheter at flow rate of 1.8 L/min, effect 1 and maximum watts of 40. This was followed by an injection of intralesional cidofovir via a 19G Wary Transbronchial histology needle. A vial of 375 mg/5 mL of cidofovir was diluted in 30 mL of normal saline. The cidofovir was injected circumferentially every 0.5 cm down the trachea, with a total of 50 injections. The procedure was performed under general anaesthesia with suspended laryngoscopy, along with an ENT team that treated disease of the cords. This regimen has been tried twice before, and on follow‐up procedure at 18 months, only 20% of his trachea was affected. The next follow‐up period was in 6 months, where a 1.9 mm cryoprobe of effect 2 was used to remove the papillomas. This was followed by an intralesional cidofovir injection via a 19G Wary Transbronchial histology needle. At this follow up, <20% of his trachea was found affected (see Fig. 2). Previous exacerbations were occurring with a frequency of 4×/12 months (2013), 7×/12 months (2014) and 5×/12 months (2015).

## Discussion

It is hypothesized that HPV infections leading to respiratory papillomatosis are acquired from an infected mother at birth during the passage through the birth canal or oral or sexual contact. HPV types 6 and 11 are frequently associated with respiratory papillomatosis. The incidence in America is estimated at 1.8 per 100,000 adults [1]. This is mostly a benign condition that affects the larynx, occasionally extending below the vocal cords into the tracheobronchial tree. Rarely, it involves the lungs in 2–5% 1. Prolific papilloma growth in the lower respiratory tract results in: post‐obstructive pneumonia, abscesses formation and respiratory failure. Long‐term effects include parenchymal lung destruction and chronic respiratory failure.

Factors associated with aggressive disease include: HPV 11 subtype, early spread to subglottis, onset before age 3 and concurrent viral infections. Roughly 3–5% of lesions undergo malignant transformation 1. Risk factors for this include: smoking, excessive alcohol consumption, radiation therapy, immunosuppression and chemotherapy. This condition is diagnosed on laryngoscopy +/− bronchoscopy, depending on disease extent, for direct visualization and confirmatory histological and microbiological diagnosis on resected papillomas.

Treatment includes surgical excision or ablative procedures for debulking of airway obstruction while preserving normal structures. In recent times, for patients who require surgical intervention >4 times a year, adjuvant therapy is suggested.

The most well‐proven adjuvant therapy is cidofovir (antiviral). Cidofovir is injected intralesionally at the base of the papilloma once resected. It works by incorporating into the virus DNA chain and inhibiting viral DNA polymerization and hence, replication. Cidofovir’s side effects include nephrotoxicity, hepatitis, and neutropenia. Of note is a Portuguese case study, in which all four subjects with extensive lesions who had previously tried multiple management options were retrospectively analysed 2. At 6‐year follow up, complete remission was achieved after five administrations of locally injected cidofovir after YAG laser. However, two of four patients had malignant transformation to epidermoid carcinoma, and one of four had severe dysplasia on histology. Other case series have reported partial to complete remission without any side effects in 24 of 26 patients at 6 months and 15 of 17 subjects at 1 year, respectively 3, 4. A systematic review in 2013 concluded that there is insufficient evidence from controlled trials to make reliable conclusions 5. Notably, the dose, concentration, and frequency of administration were different among all case series. Also noted is the lack of comparison group, blinding, specification of disease severity and differing treatment protocols. Therefore, conclusions are cautiously accepted.

In conclusion, recurrent respiratory papillomatosis is a rare condition that can extend to affect the large, and rarely small airways. This case report demonstrates the success of cidofovir as an adjunct therapy in prolonging symptom remission in RRP involving the upper respiratory tract. There remains a need for future robust clinical trials, with long term follow‐up to accurately assess the efficacy and side effects of cidofovir to allow its acceptance in RRP therapy.

### Disclosure Statement

Appropriate written informed consent was obtained for publication of this case report and accompanying images.
